# Personalized medicine in colorectal cancer 

**Published:** 2020

**Authors:** Padina Vaseghi Maghvan, Shabnam Jeibouei, Mohammad Esmael Akbari, Vahid Niazi, Farshid Karami, Alireza Rezvani, Nafiseh Ansarinejad, Masroor Abbasinia, Gisoo Sarvari, Hakimeh Zali, Ramin Talaie

**Affiliations:** 1 *Proteomics Research Center, Shahid Beheshti University of Medical Sciences, Tehran, Iran*; 2 *Department of Biotechnology, School of Advanced Technologies in Medicine, Shahid Beheshti University of Medical Sciences, Tehran, Iran*; 3 *Cancer Research Center, Shahid Beheshti University of Medical Sciences, Tehran, Iran*; 4 *Department of Tissue Engineering and Applied Cell Sciences, School of Advanced Technologies in Medicine, Shahid Beheshti University of Medical Sciences, Tehran, Iran*; 5 *Department of Hematology, Medical Oncology and Stem Cell Transplantation, Hematology Research Center, Shiraz University of Medical Sciences, Shiraz, Iran*; 6 *Department of Hematology and Oncology, Iran University of medical science, Tehran, Iran*; 7 *National Iranian South Oil Company, Ahvaz, Iran*; 8 *Department of Pharmaceutical Chemistry, Faculty of Pharmaceutical Chemistry, Tehran Medical Sciences, Islamic Azad University, Tehran – Iran*; 9 *Gastroenterology and Liver Diseases Research Center, Research Institute for Gastroenterology and Liver Diseases, Shahid Beheshti University of Medical Sciences, Tehran, Iran*

**Keywords:** Colorectal cancer, Personalized medicine, Biomarker, Targeted therapy, Chemotherapy

## Abstract

Colorectal cancer (CRC) is a heterogeneous disease with various genetic and epigenetic factors leading to difficulties in response to both the therapy and drug resistance. Moreover, even in tumors with similar histopathological characteristics, different responses and molecular features could be observed because of the genetic basis and its interactions with the living environment. Through personalized medicine, we can classify patients into separate groups according to their genetic and epigenetic features and their susceptibility for a specific disease which could help with choosing the best therapeutic approach. In this review, genetic and epigenetic factors that cause heterogeneity in colorectal cancer are evaluated and proper drug administration in both chemotherapy and target therapy are suggested.

## Introduction

 Medical and biological sciences have shown that despite similar phenotype and pathology of a certain disease, etiology is different in patients and patients might respond differently to a single treatment. Regarding this, the variable molecular basis that contributes to different outcomes was detected. Notably, only 25% of cancer patients have responded to selective treatments. Genetic and epigenetic alterations are the main cause of hereditary and sporadic form of CRCs(1). Factors involved in personalized medicine of colorectal cancer include patient’s characteristics and unique properties of tumors that provide information about the prognosis. Source of high heterogeneity in CRC patients is genetic and epigenetic alterations such as CIN, MSI and CIMP. These changes could occur individually or concurrently , which brings about inconsistency in tumors. Another source of heterogeneity in CRC is the signaling pathways. Change in Wnt, RAS-MAPK, OI3K, TGF-β, and P53 in the marker of mutations in critical genes in CRC. Host-tumor interactions are additional source of heterogeneity in individuals affected by this disease. It is believed that these interactions are highly dependent on the genetic composition of normal cells. Therefore, clinical manifestations will occur as the result of the genetic background and genomic changes. In addition to all these factors, tumor microenvironment, background diseases, hormonal alterations, stress, nutritional diet, and lifestyle impact disease heterogeneity. 

**CRC Heterogeneity**

Two types of heterogeneity including inter-tumor (between the subtypes of CRC) and intra-tumor (between the tumors of one person with cellular heterogeneity) lead into different prognosis, drug resistance, and challenges in selecting the optimum treatment (2-7). Heterogeneity in CRC has been discussed in terms of local and systemic variables. Host-tumor interactions play a role as a key variable not only in the tumor but also in the whole organism. These interactions are strongly related to the genetic composition of a normal cell, hence different clinical manifestations are seen in the affected person, which is hidden in their genetic background(8). Tumor’s micro environment with variable cells are major variables between the patients(9). Immunological heterogeneity in CRC is an important issue that more than one decay is in challenge and different criteria until now was approved i.e., MHC antigens alteration enable colorectal cancerous cells to be hidden from immune cells(10) or another achievement was the effectives of anti PDL1 drug? on the specific type of patients, MSI subtype(11). Metastatic patients also show all these heterogeneities(12). Cell-to-cell heterogeneity in one tumor probably has a developmental-like mechanism or occurs in response to an exogenous factor like a drug. Researches have shown that this type of heterogeneity plays a vital role in drug resistance and disease occurrence, and relapse as a consequence(13). Association between heterogeneity and genetic variation changed the normal biological process and signaling pathways in cancerous cells. The main signaling pathway in normal colorectal tissue is Wnt associate with promoting cell proliferation and mutation in downstream molecules. It may cause the inactivation of APC and activation of β-Catenin and enter the cell to tumorigenesis process(14). Wnt pathway with about 93% change undergoes most changes in this disease and tumors have shown disorders in this pathway, regardless of their mutation rate (low or high)(9). Carcinogenesis process of colorectal tissue promote by different bypass pathways that cell growth, proliferation and differentiation. They are receptor tyrosine kinase (RTK) signaling, TGFβ and TNF-α signaling. Receptor or downstream molecules in these pathways is targeted for therapies(15). Besides, tumors with high mutation rate are rich in genetic alterations in TGF-β pathway, while tumors with low mutation level are primarily affected in the P53 pathway (9). Based on transcription profile, tumors are divided into three groups of MSI/CIMP, CIN, and aggressive mesenchymal phenotype. Importantly, primary pathways of MSI and CIMP are dependent on epigenetic changes. Thus, epigenetics is one of the causes of heterogeneity in CRC(6). MicroRNA molecules are the other regulatory biomarkers reported in angiogenesis, disease relapse, and mortality rate. They could work as a reliable tool for diagnosis and therapy (16, 17), i.e., association between miR-19a expression level and poor prognosis or significant correlation of cluster miR-17-92a expression level with relapses in CRC patients (18).

**Prognostic and predictive biomarker in CRC**

Prognostic and predictive biomarkers are defined as patient selection factor for treatment type and evaluation of the treatment on patients, respectively (19). The most important causes of heterogeneity in CRC used as molecular markers to optimized and tailored treatment regimens and predict the prognosis i.e. MSI and KRAS hopefully become biological prognostic and/or predictive markers. Most diagnosis criteria do not have adequate sensitivity and accuracy regarding multiple studies on the role of KRAS demonstrated that the prognostic indicator of specific mutation type of KRAS or detected only in some stages or recurrence with other abnormalities like p53 mutation (20-22). With conflicting results of conventional biomarkers in CRC, new studies are needed to select the best therapy or predict prognosis . In addition to many detected biomarkers detected, some of which applied in clinic, there is a large number of biomarkers to be approved in CRC. Recently, great efforts have been made to identify novel biomarkers for more effective treatment of the colorectal cancer patients. Three of these markers are telomerase length, telomerase activity and micronuclei frequency(23) which have been proposed as new potential biomarkers to ascertain the prognosis of the disease and predictive respond to treatments. The critical domain in personalized medicine is disease relapse prediction. Several studies have been conducted to find biomarkers for CRC relapse prediction. The expression level of a gene such as *MACC1* or micro-RNAs like miR-19a might probably be helpful for this prediction (18), which require further studies. Table 1 demonstrates prognostic and predictive biomarkers in CRC.

**Drugs**

**Figure 1:**
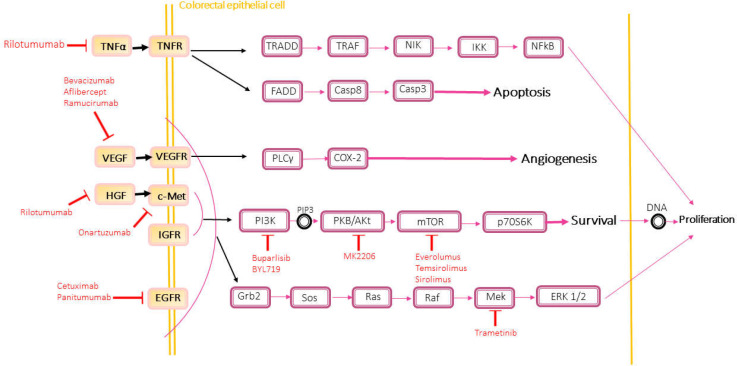
All cell signaling pathways blocked by targeted drugs such as Cetuximab which is a monoclonal antibody for blocking epidermal growth factor receptor. Thus, survival, proliferation, migration, invasion, and angiogenesis of cancer cells can be inhibited. Resistance to these drugs is as a result of a mutation in one of the involved genes in these cell signaling

Personalized medicine offers the most efficient therapeutic process for the patients. All the targeted therapy programs presented for CRC refer to the association of critical genes to the central mechanisms of disease progression, as represented in Figure1, all pathways that could be repressed by the targeted drugs to inhibit the proliferation and invasion. However, more research is needed to investigate drug resistance mechanisms in distinct groups of CRC to understand probable prognosis in different categories of the patients. Chemotherapy compositions have been regarded to have had more challenges in recent years. Because of the judgment of CRC patients’ prognosis nowadays is restricted to the limited information based on the high or low frequency of MSI, patient’s MSS status, mutations in BRAF or PIK3CA genes, all of which influence in the identification of the potential tumors’ invasion rate and differentiation, good or poor prognosis, and the degree of progression, and sometimes the tumor’s location. Here two categories of treatment in CRC will explain in details the pharmacogenetics used in clinical trials or in researches.

- **Drugs Related to Targeted Therapies**

**1.Bevacizumab:** this drug is a monoclonal, recombinant human IgG1-antibody that acts against the *VEGF-A* ligand and is related to *VEGFR1* and 2 receptors. In 2006, FDA approved Bevacizumab combinatorial drug based on 5-Fluorouracil as the second-line treatment for mCRC. This drug is prescribed because of the significant results of OS measure improvement in patients receiving Bevacizumab in addition to FOLFOX4. Furthermore, US food and drug administration approved Bevacizumab in combination with Fluoropyrimidine-Irinotecan or Fluoropyrimidine-Oxaliplatin for mCRC patients who have received Bevacizumab in the first-line of treatment, and had progressive disease. Thus, patients were given a new regimen depending on their previous treatment regimen. A significant improvement was seen in OS and PFS indicators of patients who had received Bevacizumab along with the described chemotherapies (24). In addition to metastatic colorectal cancer, this drug is used to treat lung cancers, glioblastoma, and renal cells carcinoma(25).

Variations in *VEGFA* gene are shown to alter sensitivity to bevacizumab. rs2305948 SNP in *VEGFR2* has been associated with diminished bevacizumab treatment outcome. In a study on cancer mCRC patients who were treated with bevacizumab, researchers observed increased PFS/OS associated with rs699947, rs833061, rs2010963, rs3025039 SNPs in *VEGFA(*26*). *A large cohort study on mCRC patients revealed an increased risk toxicities associated with a treatment regimen based on first-line 5-flourouracil and irinotecan plus bevacizumab(27). Recently, it has been determined that bevacizumab and alternative splicing of VEGFA could be predictive and prognostic biomarkers respectively, in patients treated with irinotecan-based chemotherapy and bevacizumab respectively (28).

**2. Ramucirumab:** This drug is also a monoclonal human IgG1 antibody similar to Bevacizumab which is classified in anti-*VEGFR* drugs group, and inhibits *VGFR2* thereby preventing the progress of signaling pathways for angiogenesis, division, and survival(29). In 2015, FDA approved this drug in the second-line treatment in combination with FOLFIRI for treating mCRC patients. The mentioned patients have received Bevacizumab with Oxaliplatin and Fluoropyrimidine in the first-line treatment but were still experiencing disease progression. The treatment regimen of Ramucirumab accompanied by FOLFIRI leads to the improved OS and PFS rates (30). Unfortunately, no biomarker has yet been identified for choosing the patients to respond to this drug. This drug is also used for gastric and lung cancers, and since CRC treatment with this drug does not last long, no unique mechanism is found for the resistance to this drug(31).

**3. Aflibercept:** This drug which is also known as Zaltrap is an inhibitor of the receptor protein bound to angiogenesis ligands including *VEGF-A, VEGF-B*, and *PLGF* to prevent the progression of angiogenesis signaling pathways. In 2012, Zaltrap regimen along with FOLFIRI was approved by the US for patients who were resistant to regimens containing Oxaliplatin, because the research emphasized improved OS in patients accepting this strategy(32). Mechanism of resistance to this drug and other anti-*VEGFR *drugs are alike and still unknown. As shown in the researches, resistance to *VEGF*-Trap treatment leads to an increased expression of *VEGF-C* which bestows the improvement of alternate angiogenesis pathways for adaptation against the drugs(33).

**4. Regorafenib:** This drug acts by targeting several protein kinases in angiogenesis pathway associated with *VEGFR1, VEGFR3, VEGFR2*, and *TIE2*. Its oncogenic targets include *KIT*, RET, BRAF, and *BRAF-V600E*. *PDGFRα* and *β* are tumor microenvironment targets and*p38 MAP kinase*, *FGFR1* and 2 are other tyrosine kinases that are targets for this drug(34). This drug was approved in 2012 for mCRC patients who had previously used regimens based on Oxaliplatin, Irinotecan, or Fluoropyrimidine or had received anti-VEGFR and anti-EGFR regimens(35). Since the extent of inhibition is immense, the resistance mechanism is too complicated and still unexplained. Some researchers state that overcoming the resistance requires extensive changes. Hence, the Notch signaling pathway has attracted them. This pathway regulates different cellular processes such as proliferation, differentiation, and apoptosis so that it could be a way for resistance to various targeted treatments. In research on colorectal cancer cells resistant to Regorafenib, a high level of *Notch-1* expression and transcription factors like *HEY1* and *HES1* has been observed. Invasiveness of those colorectal cancer cells resistant to Regorafenib reduced the following *Notch-1* knockdown (36).

**5. Cetuximab:** This drug belongs to the anti-*EGFR* treatments group and is a human recombinant monoclonal IgG1 antibody that acts by blocking the epidermal growth factor receptor. Blockage of this receptor prevents progression of signaling pathways involved in cellular survival, angiogenesis, and invasion. Several studies issued in US food and drug administration approved this drug. It is utilized either in a combinatorial form or as an individual drug(37). 

Somatic mutation in *EGFR* is one of the resistance mechanisms, in which the replacement of serine by arginine in codon 492 (S492R) affects the external domain of this receptor, and causes resistance to Cetuximab drug(38). Researches have shown that this somatic mutation is acquired after receiving anti-*EGFR* drugs(39). According to molecular analyses, the mutation in *BRAF* and *KRAS* genes leads to resistance to anti-*EGF* agents. In addition to these genes, the mutation in *NRAS* (a membrane GTPase enzyme which is mutant in 2-5% of the mCRC patients) is effective in causing resistance to Cetuximab and its peer, Panitumumab(22). However, some patients are still resistant to anti-*EGFR* drugs without mutation in *KRAS* and *BRAF* genes. Several experiments have been performed to clarify this issue. For example, it was observed that those individuals who have a mutation in exon 20 of their *PIK3CA* gene imply resistant to Cetuximab(40). On the other hand, some researches mention mutation, lack of expression or hyper methylation of the *PTEN* gene linked to unresponsiveness to Cetuximab(41). It is also stated that patients without a mutation in KRAS and *BRAF* genes encounter enhanced *HER2* gene in their samples. Researchers believe that anti-*HER2* method could be beneficial for this subtype which is also known as *HER2*-therapy(42). Another extensive research on over 3000 mCRC patients has also confirmed that more investigations are required to prove the correlation between *HER2* overexpression and resistance to anti-*EGFR* therapeutic strategy or disease relapse(43). *MET* or hepatocyte growth factor receptor (a tyrosine kinase involved in cell division and apoptosis) could be mentioned among resistance factors to anti-*EGFR* strategy. It is suggested that MET has the potential to activate *PIK3/PKB* pathway independent from *RAS* and could interfere with anti-*EGFR *strategy(44). High *IGF1R* expression has also been recognized in 50 to 90% of CRC patients and plays a vital role in oncogenic changes and cancerous cells growth and survival, and is indicative of poor prognosis and resistance to anti-*EGFR*(45, 46).

**6. Panitumumab:** This drug is also among epidermal growth factor receptor inhibitors like Cetuximab. It belongs to the human monoclonal IgG2 antibodies. The effect of this drug on OS and PFS rates is similar to Cetuximab; furthermore, the failing of its effects when patients encounter with a mutation in the *KRAS* gene have been ascertained(47). Mechanism of resistance to Panitumumab is comparable to resistance mechanisms for Cetuximab; the only difference is the observation of acquired mutations in *EGFR* after treatment with Cetuximab but not for Panitumumab(39).

Targeted drugs related to epithelial cell signaling pathway from CRC mentioned above are shown in Figure 1.

**B) Chemotherapies**

**Figure 2:**
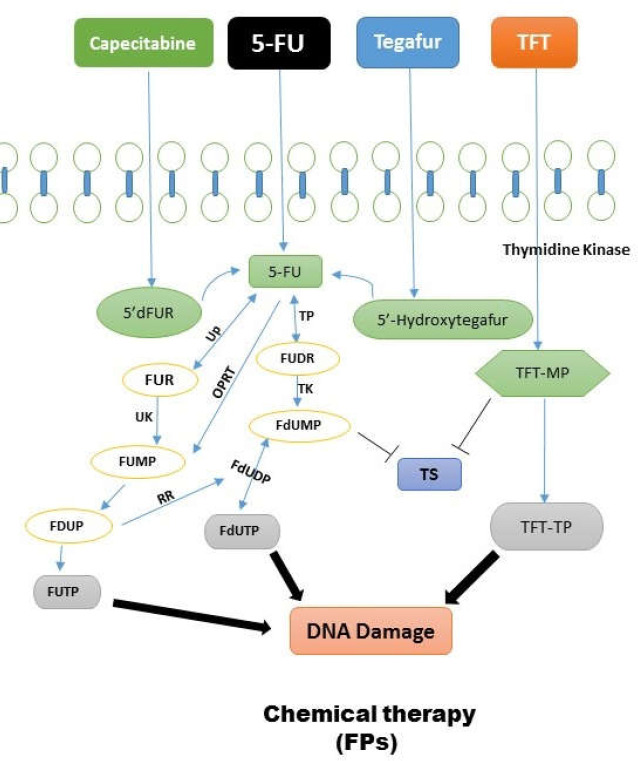
Fluoropyrimidines family cause cellular death through inducing disturbance in DNA duplication and transcription

**1. Fluoropyrimidines (FPs) family:** 5-FU and Capecitabine are part of this group. These drugs provoke cellular death through inducing disturbance in DNA duplication and transcription. Mutation in MMRs and hyper methylation of promoters of mentioned genes could make resistance to this group of drugs(48). 5-FU is a drug from FPs class that has extensive application in colon cancer treatment. This compound as a synthetic fluorinated pyrimidine analog is administered through venous injection. It has several metabolites including FdUMP, 5-FdUTP, and 5-FUTP. This drug tends to produce fluorinated nucleotides by different mechanisms and inserts them in DNA structure instead of thymidine, thereby disrupting duplication and ultimately causing cellular death (see Figure 2). The primary active metabolite of this drug, FdUMP, inhibits *TS* enzyme, hence preventing dUMP conversion into dTMP and interfering with cell cycle in the cancerous cell(49). *TS* enzyme is coded by *TYMS* gene, and the studies designated that an increase in the expression of this gene reduces the effects of this drug or drives resistance to 5-FU(50). In some other studies, it has been mentioned that diminished expression of *MTHFR* enzyme which is the regulator of cell folate results in elevated inhibition of *TS* enzyme, enhancing 5-FU drug efficacy(51). Therefore, the SNPs of this enzyme that reduce its activity and causes drug sensitivity were evaluated. In fact, this issue is still under study and has not been approved entirely. Converting 5-FU to the active metabolite needs 5-FdUMP, *TP* enzymes which are encoded by the *TYMP* gene. Some investigations showed that high *TP* levels lead to sensitivity to this drug. Besides, it is declared that this enzyme is involved in metastasis and angiogenesis enhancement(52). OPRT is also another enzyme that plays an essential role in converting 5-FU into the active metabolite, and it is encoded by the UPMS gene. High expression of this gene and the subsequent elevation in *OPRT* enzyme level is accompanied by sensitivity to 5-FU. However, more studies are required to prove these findings(53).

Some in vitro studies on malignant cells detected high expression of an enzyme encoded by the* DPYD* gene which is responsible for 5-FU catabolism. Indeed, this mechanism leads to the elimination of drug efficacy. Choosing this enzyme as a marker for resistance to 5-FU requires more clinical evidence. Generally, it can be stated that alterations in *DPYD, MTHFR, TYMS*, and *UPMS* genes could be potential targets for combatting resistance to 5-FU(52-54). Capecitabine belongs to FPs family and is administered orally. This drug finally converts into 5-FU in tumor cell through specific steps. Thus, it seems that the resistance mechanism for this drug is like 5-FU; high *DPD *expression is related to resistance to this drug and raised TP expression causes more sensitivity to it(48). Tegafur is also an oral FP like Capecitabine and finally changes into Fluorouracil. Cytochrome P-450 is required to metabolize this drug and is coded by *CYP2A6*. Researches show that patients whose *CYP2A6* gene is wild-type were more sensitive to the drug. Polymorphisms probably involved in the reduced metabolization of the drug are also under examination(55).

Trifluridine/Tipiracil or TAS-102: In 2015, this compound drug earned approval for metastatic colorectal cancer patients. These patients had already used anti-*EGFR* or anti-*VEGFR* drugs or chemotherapy regimens containing Irinotecan, Oxaliplatin or Fluoropyrimidine. Both OS and PFS values improved after taking this drug. It is administered orally and contains a Fluoropyrimidine part. Phosphorylation of this part (*TFT*) by thymidine kinase inhibits *TS* enzyme similar to other FPs.

Moreover, *TFT* with three phosphates (*TFT-TP*) is inserted into DNA structure and disturbs duplication. It has probably a resistance mechanism similar to FPs. Resistance mechanism for this drug has not currently been defined(48).

**Figure 3:**
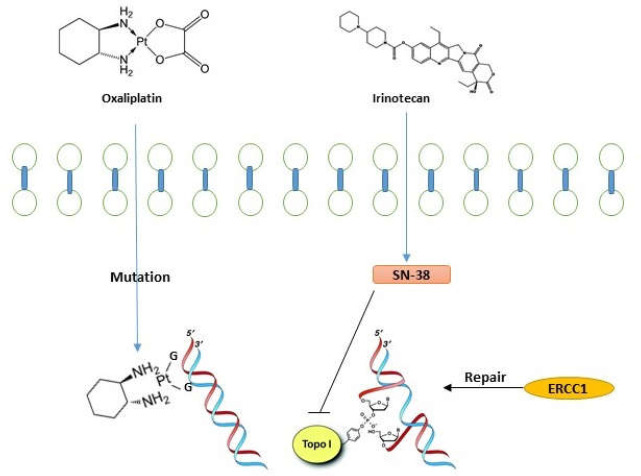
Oxaliplatin is a compound that produces cross-linkages in DNA. This defect can be repaired by MMR system and NER mechanism. Irinotecan effect on duplication and transcription by inhibiting topoisomerase I enzyme

**2. Oxaliplatin:** This drug is a platinum compound that produces cross-linkages in DNA by DNA-Pt adduct (see Figure. 3). 

**Table 1:** Prognostic and predictive biomarkers in CRC

	Tissue derived	Blood derived
Prognostic biomarkers	MUC2, SATB2, CK20/CDX2	preoperative CEA
	VEGF, Imp3, TNIK, KRAS, NRAS	postoperative CEA
	P53, BRAF, miRNAs, MSI	CA19-9, CTC
Predictive biomarkers	KRAS/NRAS, BRAF, PI3K	MSI, CD133

Afterwards, the MMR system identifies these defects and induces apoptosis in cells. Repair of DNA damages through the NER mechanism could explain chemotherapy failure. High expression of *ERCC1* and *ERCC2* proteins which are involved in this mechanism is seen followed by a weak response to Oxaliplatin-based regimen. Furthermore, numerous polymorphisms in *ERCC1* gene could be a suitable prediction candidate for response to treatment(52, 56). On the other hand, BRCA1 protein has an essential role in repairing the DNA cross-links, and any kind of change in this gene or its epigenetic knockdown which rarely occurs in CRC is indicative of sensitivity to Oxaliplatin. The interaction between *BRCA1* gene products and *SRBC* gene determines the role of this factor in responding to this drug, because it has been perceived that elevated *SRBC* expression accompanies with sensitivity to the drug. Besides, it seems that *SRBC* is involved in trafficking of intracellular vesicles (57, 58). Efflux of drug from the cell is another theory for explaining drug resistance. Combination of Platinum with glutathione (*GSH*), an antioxidant molecule which prevents nucleic acid oxidative damages, causes drug efflux from the cell by *ABC* transporter proteins. Investigations showed that high *GSH* expression levels in tumors induce resistance to Platinum(59).

**3. Irinotecan:** This drug interferes with DNA duplication by inhibiting topoisomerase I enzyme, thereby causing cell death (see Figure 3). This drug acts better when combined with FPs and produces a remarkable improvement in OS and PFS values. The active metabolite of this drug (SN-38) reversibly forms a complex with *TOPO I* and DNA. Any alteration in the activity of this complex decreased *TOPO I* expression, or low SN-38 level could make resistance to Irinotecan. Cytochrome *P-450* enzymes convert Irinotecan into an inactive metabolite, and then carboxylesterase 1 and 2 produce SN-38 with hydrolyzing. Some studies revealed that carboxylesterase activity is correlated with drug sensitivity(60). Besides, drug efflux by *ABC *proteins creates resistance to this drug. Polymorphisms in transporter proteins explain a variety of toxicity in patients(61, 62). Researches have shown that *TOPO 1* expression rate or copy numbers could be useful in drug resistance, meaning that response rate to the mentioned drug is directly correlated with *TOPO 1 *gene copy numbers. Point mutations in *TOPO1* gene which damage its binding sites with SN-38 also cause drug resistance(63, 64). The major enzyme responsible for glucuronidation of SN-38 is UGT1A1 which metabolizes and detoxifies irinotecan. FDA of the US has approved this as a method for the prediction of irinotecan-related acute diarrhea and neutropenia.

*To increase the efficacy and patient’s safety, UGT1A1* genotyping was initiated for dose-escalated irinotecan in mCRC. Patients with homozygous UGT1A1*28/*28 are more vulnerable to irinotecan, while patients with heterozygous UGT1A1*1/*28) encounter are exposed to an increased risk of irinotecan toxicity and patients with the homozygous UGT1A1*1/*1 genotype are more resistant to irinotecan(65).

**Future perspective**

Since personalized medicine works on three subjects of determining disease indices in people, choosing the best therapeutic method and predicting disease relapse, it seems that regarding colorectal cancer, more researches are required in order to achieve favorable results. For determining the indices of familial colorectal cancer, familial background can be traced. Considering the most common inherited colon cancer which is known as Lynch syndrome, the presence of people affected by this disease in the family and follow-up of PMS2, MSH2, MSH6, and MLH1 genes might be helpful. The index for familial adenomatous polyposis (FAP) is also a mutation in the APC gene. Reports of the possible role of other genes such as KIF23, CENPE, MUTYH, POLE, and POLD1 in genetic predisposition for CRC and presence of a mutation in specific genes in populations affected by CRC are all suggestive of unidentified features of personalized medicine for colorectal cancer. Much needs to be clarified regarding the indices of sporadic colorectal cancer that may firstly result in early diagnosis of the disease and then the patients’ targeted therapy. Although many mutations and signaling pathways involved in the disease have been identified, an efficient index for predicting the sporadic type of this disease has not yet been introduced. The reason for this is the variety in genetic and molecular details of the mentioned disease. The role of studies based on personalized medicine for this disease is irrefutable. The overall results of these researches could help efficient classification of CRC patients because the existing classifications have several deficits. 

Regarding the variety in response to drugs for colorectal cancer, the need for more researches about patient’s classification is being felt. Various drug resistances in these patients demonstrate that molecular information available about this disease is insufficient for understanding its complexities. Consequently, additional studies about all the individual mentioned subjects in the drug resistance factors could be carried out. Extensive studies on personalized medicine in breast cancer treatment have led into specific classification for this disease and treatments are more targeted and efficient than before. Thus, mortality rate and drug resistance during treatment have reached the minimum levels. Systemic and comprehensive genomics, proteomics, and metabolomics studies along with bioinformatics evaluations are of great benefit.

Patient derived xenografts (PDX) are models of cancer applied clinically and do not determine all limitations, but the number of them is the insufficient amount of fresh tissue in some case or the model not established for all patients**.** Data analysis from samples used for drug screening by PDX and tumor organoid represents a similar result. Genomic analysis between native tumor tissue and both models show nearly complete concordance.

This study sheds light on the integrated data obtained from methods, including genetic screening, bioinformatics algorithms and PDX, and organoid models might be helpful in diagnosis and proper drug administration in both chemotherapy and target therapy to predict the prognosis.

## Conclusion

With all chemotherapies or targeted therapies in colorectal cancer, the most critical factor in treating patients is to focus on early diagnosis and development of studies that work on early diagnosis biomarkers. Pharmacogenomic and pharmacogenetics discoveries have recently begun necessitating the inclusion of various populations. All of which requires the development of molecular methods, availability, and low cost. On the other hand, regardless of sophisticated molecular methods, it is possible to find the effective drug and the appropriate dosage for the patient's tumor spheroids, solely by cellular methods and focusing on heterozygous tumor cells in the presence of various therapeutic regimens in the laboratory.
